# Two-Phase Approach for Fast Topology Optimization of Multi-Resonant MEMS Involving Model Order Reduction

**DOI:** 10.3390/mi16040401

**Published:** 2025-03-29

**Authors:** Siyang Hu, Billy Manansala, Ulrike Fitzer, Dennis Hohlfeld, Tamara Bechtold

**Affiliations:** 1 Department of Engineering, Jade University of Applied Sciences, Friedrich-Paffrath-Str. 101, 26389 Wilhelmshaven, Germany; siyang.hu@jade-hs.de (S.H.);; 2 Institute for Electronic Appliances and Circuits, University of Rostock, Albert-Einstein-Str. 2, 18059 Rostock, Germany

**Keywords:** topology optimization, model order reduction, multi-resonance, resonance frequency optimization, MEMS resonator, commercial solver

## Abstract

In this work, we propose a two-phase approach for a fast topology optimization of multi-resonant MEMSs. The approach minimizes the computation effort required to achieve an optimal design. In the first step, we perform a pre-optimization using bi-directional evolutionary structural optimization (BESO). We found in previous research that BESO can achieve optimal MEMS designs in a significantly lower number of iterations when compared to classical density-based methods. However, we encountered convergence issues with BESO towards the end of the optimization. Therefore, we introduced a second, density-based optimization phase to circumvent this issue. Finally, we introduced model order reduction to reduce the optimization time further. The novel approach is benchmarked with the design task of two common multi-resonant MEMS devices: a linear gyroscope and a micromirror. We show that the two-phase approach can achieve an optimal design within 200 iterations. With the addition of MOR, the computation of the goal function can be further reduced by 50% in our examples.

## 1. Introduction

Microelectromechanical systems (MEMSs) play an important part in our everyday life in different applications across a number of fields, e.g., telecommunications, consumer electronics, mobility, building automation, or healthcare. Their importance is expected to further increase at a growing rate due to the current development of the Internet of Things and portable/wearable electronics [1]. Many MEMS devices are resonant structures that are operated in resonance to realize their functions. These functions include sensing [2], actuation [3], timing [4], or filtering [5], with the very first application being reported in [6]. Since then, MEMS resonators have become prevalent in these aforementioned applications. This is mostly due to the possibility of fabricating these structures in batches with low-cost fabrication, similar to techniques used for integrated circuit manufacturing. Moreover, this similarity enables solutions for combining and integrating MEMSs with integrated circuits into a system on a chip. This leads to systems with superior form factors, robustness w.r.t. electromagnetic compatibility, and lower parasitic capacitances in the electrical connections between the involved components [7].

The design of MEMS resonators addresses their resonant modes and corresponding frequency characteristics. This includes tuning desired resonant modes to desired frequencies and suppressing undesired resonant modes. However, the devices’ resonance frequencies can often not be chosen independently, and geometric design alterations affect multiple resonance frequencies at once. In general, the field of MEMSs is an interdisciplinary field. MEMS devices intrinsically involve multiple physical domains, requiring different types of representation with varying levels of abstraction, making the design of MEMSs a highly complex matter. As reported in [8], the MEMS design process is still mostly performed in a “trial-and-error fashion”. It often relies on an expert’s experience with similar devices to find a suitable layout. This motivates a more systematic approach for identifying design geometries with desired properties, to increase the effectiveness and efficiency of the design process.

A systematic approach to geometry alteration is provided by mathematical optimization. It alters a design based on a user-defined objective function under given constraints. Typically, the layout of MEMSs is composed of simple geometrical substructures, such as rectangular mass blocks and flexible springs in the form of cantilevers or folded beams etc. Therefore, design optimization of MEMSs mainly consists of altering the geometric dimensions of these substructures. Due to the complex design space inherited from the complexity of the MEMS design process, heuristic approaches are preferred to traditional optimization algorithms [9]. Ref. [9] provides an overview of evolutionary design optimization approaches, which are predominant in the field of MEMSs. In [10,11], the authors propose an efficient, optimization-based framework for the design of MEMS gyroscopes and other inertial devices. The framework incorporates a combination of sub-structuring and static model reduction techniques. Recent studies on the optimization of scanning mirrors can be found in, e.g., Refs. [12,13], focusing on different aspects of the design, such as driving torque and crosstalk during actuation or dynamic deformation.

However, optimizations based on geometrical dimensions are strongly limited by the initial design, and the composition of simple geometric substructures will remain, although in optimized dimensions. More design freedom can be provided for topology optimization (TO). Instead of altering geometric dimensions, TO optimally distributes material onto a fixed design space. Thus, in theory, any shape or form can be achieved. This material distribution method was first introduced in [14]. In [15], the popular solid isotropic material with penalization (SIMP) method was introduced. The SIMP method assumes an isotropic material that is to be distributed over a finite-element (FE) domain. It introduces elemental pseudo-densities for each element relative to the density of the isotropic material as a design variable. This design variable determines the Young’s modulus and density via the respective power-law interpolation scheme. For SIMP-based TO, the method of moving asymptotes [16] or the optimality criteria (OC) method (e.g., [17]) is typically incorporated to find an optimum. An alternative approach to TO is evolutionary structural optimization (ESO) methods. The first examples of ESO can be found in [18,19] or [20]. ESO achieves optimal design by removing inefficient elements according to some predefined measure. The method was later extended to bi-directional ESO (BESO) in [21], which allows elements to be reintroduced. An overview of most state-of-the-art TO approaches is presented in [22,23], in which they are reviewed and compared. Some of the most recent studies have also introduced artificial intelligence (AI) to the field of TO. A comprehensive review of TO via machine and deep learning is provided in [24].

Even though TO was originally designed for compliance minimization (as shown in, e.g., [14]), it has proved effective in a plethora of applications across all fields of engineering, e.g., in aerospace engineering [25] or in the field of meta-materials [26]. The first relevant works, w.r.t. the field of MEMSs in the form of multi-frequency optimization, can be found in, e.g., [27,28,29] (SIMP) or [30] (BESO). A review discussing TO for vibration problems can be found in [31]. Early application of TO in actual MEMS devices can be found in [32,33]. More recent works have shown successful TO of many related devices: In [34,35], the authors consider three different optimization formulations for the optimization of MEMS gyroscopes to achieve targeted resonance frequencies; Ref. [36] proposes a TO to improve the dynamic stability of a resonant MEMS scanner used for LiDAR applications, and in [37], TO is performed on a multi-resonant piezoelectric energy harvester to maximize its power output for a predefined frequency range.

Because of the optimization problems posed by MEMS design, TO may require very high computational effort, especially when broadband frequency responses or transient behaviors (due to parasitic effects) and fine resolutions are required. This is why model order reduction (MOR) has been introduced to remedy this issue. MOR replaces the original high-fidelity model with a significantly lower-dimensional but still highly accurate surrogate. Thereby, it can reduce the computation time required for model evaluation by several magnitudes [38]. An up-to-date overview of classical MOR in TO is provided in [39]. Some recent studies also suggest using AI to reduce the computational burden of TO. Ref. [40] proposed a deep learning-based MOR that allows TO to be performed on coarser meshes while obtaining similar results compared to traditional methods on finer meshes.

In this work, we explore a two-phase TO approach for multi-resonant MEMS applications. The two phases combine an initial evolutionary TO with a subsequent density-based TO. Aside from its compatibility with commercial FE software [23], we found in our prior work that BESO can achieve an optimal design, requiring a significantly smaller number of iterations compared to density-based methods. However, in our previous work, we encountered convergence issues during BESO of resonant structures. This is why we introduce a second, density-based optimization phase to circumvent this issue. Such an approach has been discussed in the community; however, to our knowledge, it has yet to be implemented and evaluated. The only other work combining SIMP with BESO we are aware of is [41]. The aforementioned work is published in the field of multi-scale TO for lightweight cellular material, where BESO is applied to the optimization of the microstructure while SIMP is simultaneously applied to the microstructure.

The two-phase workflow is designed in such a way that the major optimization task is carried out during the BESO phase in a small number of iterations. Thereafter, a second optimization phase using density-based methods is performed to achieve algorithmic convergence. Since we aim to reduce the computational effort to a minimum, we also introduce MOR techniques as an additional measure to achieve this goal. Note that in our implementation, MOR is only considered in the density-based TO phase. This is because the commercial FE solver can provide all the required information during the BESO phase.

For the evaluation of the two-phase approach, we choose simple and well-established MEMS structures: an MEMS gyroscope and an MEMS scanning mirror. For the design of the MEMS gyroscope, we compare the results obtained by the combined approach with the results obtained by both BESO and the density-based approaches, individually, based on their performance given a limited number of iterations.

This paper is organized as follows. In Section 2, we briefly introduce the mathematical models of MEMS resonators and their reduced-order modeling. Section 3 defines the optimization problem we consider for the design of this class of devices to achieve the desired resonant frequency and amplitudes and avoid undesired ones. This includes the definition of an objective function, constraints, and sensitivities. In Section 4, the novel two-phase approach is proposed, before it is applied to the example models in Section 5. In Section 6, we conclude the paper and give an outlook on possible future research.

## 2. Numerical Modeling of Mechanical Resonators

In this section, we briefly introduce the numerical models involved in this paper. We first describe the model we obtain from the FE method. Subsequently, we provide a short outline of the projective model order reduction that is implemented in this work to speed up the optimization.

### 2.1. Finite Element Modeling

In this work, our geometries are modeled with industrial FE software (cf Section 5), using linear 3D hexahedron elements. The spatial discretization of the governing partial differential equation results in a second-order linear dynamical system, which is described by the following system of ordinary differential equations (see, e.g., [38,42]):

(1) $$\Sigma :\left\{\begin{array}{c} M\ddot{x}+D\dot{x}+Kx=bu,\\ y=C^{T}x. \end{array}\right.$$

Here, in (1), $x\in R^{n}$ is the state vector, i.e., a vector containing all nodal displacements, and $M,D,K\in R^{n\times n}$ are, respectively, the mass, damping, and stiffness matrices of the structure. As a simplification, we set D=0, making the dynamics undamped. The remaining system matrices are assembled from respective element matrices $M_{e}$ and $K_{e}$:

(2) $$\begin{array}{c} K=\sum_{e}K_{e},\;M=\sum_{e}M_{e}, \end{array}$$

where the operator $\sum_{e}$ indicates the standard FE assembly process. *u* is the input of the system, which is the mechanical force in this case. $b\in R^{n}$ is the input vector, which distributes the force upon the structure nodes. $y\in R^{q}$ is the user-defined output of the system, gathered via the input matrix $C\in R^{n\times q}$.

### 2.2. Model Order Reduction

There are many different ways to approach the model reduction of dynamical systems. In this work, we focus on the projective approaches. Their common idea is to achieve complexity reduction by approximating the state vector with a surrogate in a significantly lower dimensional basis and then projecting all governing equations onto a compatible low-dimensional subspace [43,44]. In detail, let the columns of $V\in R^{n\times r}$ be the basis of an *r*-dimensional subspace $V\subset R^{n}$, with r≪n. An approximation of the state vector is given by $x\approx {\mathrm{Vx}}_{r}$, with $x_{r}\in R^{r}$. The residual r of this approximation with reference to Equation (1) is given by

(3) $$r=M{V\ddot{x}}_{r}+D{V\dot{x}}_{r}+K{Vx}_{r}-bu,$$

and the output equation is transformed into

(4) $$y_{r}={C^{T}\mathrm{Vx}}_{r}.$$

By enforcing the Petrov–Galerkin condition,

(5) $$W^{T}\left(M{V\ddot{x}}_{r}+D{V\dot{x}}_{r}+K{Vx}_{r}-bu\right)=0,$$

we obtain the following reduced system:

(6) $$\Sigma_{r}:\left\{\begin{array}{c} M_{r}{\ddot{x}}_{r}+D_{r}{\dot{x}}_{r}+K_{r}x_{r}=b_{r}u,\\ y_{r}=C_{r}^{T}x_{r}, \end{array}\right.$$

where the reduced matrices are computed as

(7) $$\begin{array}{c} \begin{array}{cc} \left\{M_{r},D_{r},K_{r}\right\} & =W^{T}\left\{M,D,K\right\}V\\ b_{r}=W^{T}b, & \;C_{r}=V^{T}C. \end{array} \end{array}$$

Different projective MOR approaches differ in their choice of projection matrices *V* and *W*. There are mainly three classes of approaches, approaches based on singular value decomposition (SVD), approaches based on Krylov subspaces, and approaches based on a combination of the first two. A detailed introduction of the approaches exceeds the scope of this work. Therefore, we will only give a brief overview of approaches widely used for the reduction of dynamical systems. A detailed introduction of MOR is provided in [43].

#### SVD-Based MOR

The class of SVD-based MOR methods includes widely used MOR methods such as proper orthogonal decomposition (POD), modal truncation, and truncated balanced realization (TBR). All these approaches utilize SVD to obtain their respective projection matrices—i.e., the singular vectors corresponding to the most dominant singular values are used as a projection basis. For more details, please refer to [43].

The most established SVD-based approach for the reduction of linear time-invariant dynamical systems is modal truncation. Instead of snapshot samples (POD) or Gramians (TBR), an eigenvalue decomposition is performed on the matrix pencil generated by the system matrices (K,M). The projection with the corresponding eigenbasis leads to a reduced-order system, which only retains the most dominant poles, i.e., the largest eigenvalues, of the system.

Note that when an *r*-dimensional modal truncation is applied, i.e., when the projection basis is chosen as $V=(\phi_{1},\phi_{2},\cdots ,\phi_{r})$, where $\phi_{i}$ is the *i*-th eigenvector of the system normalized by the mass matrix, i.e., $\phi_{e,i/j}^{T}M\phi_{e,i/j}=1$, the reduced system matrices become

(8) $$K_{r}=\left[\begin{array}{cccc} \omega_{1}^{2} & \; & & \\ & \omega_{2}^{2} & \; & \\ & \; & \ddots & \\ & \; & & \omega_{r}^{2} \end{array}\right],\;M_{r}=\left[\begin{array}{cccc} 1 & \; & & \\ & 1 & \; & \\ & \; & \ddots & \\ & \; & & 1 \end{array}\right],$$

where $\omega_{i}$ gives the structure’s *i*-th resonance frequencies.

Note that there are two established normalization methods for eigenvectors, unity and mass normalization. Typically, eigenvectors are normalized by the mass matrix in industrial software. Therefore, mass normalization is considered throughout this work. However, we do not expect different normalization to affect the outcome of the optimization.

Due to the simple test cases, we implemented modal truncation in this contribution. This is only to establish a procedure and showcase the potential of MOR in combination with the proposed two-phase method. We expect state-of-the-art MOR methods to provide better performance when more complex optimization problems are considered, and especially when transient behaviors need to be considered due to parasitic effects.

## 3. Optimization Problem

The major design goal of multi-resonant MEMSs is to achieve predefined resonant frequencies for desired modes and suppress undesired or parasitic resonance effects. This is mostly achieved by adjusting the design in such a way that the resonance frequencies of parasitic modes appear at higher values. The secondary objective is mostly aimed at maximizing the amplitudes of the desired modes, e.g., in the case of a sensor, the sensor response, and for energy harvesting, the power output of the device. Therefore, we formulate the general optimization problem for this class of devices as follows:

(9a) $$\begin{array}{cc} \max_{\gamma }\; & y=\sum_{i}x_{i}^{T}Kx_{i}, \end{array}$$

(9b) $$\begin{array}{cc} \mathrm{subject}\;\mathrm{to}\; & \frac{{\left(\omega_{i}-\omega_{i}^{*}\right)}^{2}}{{\omega_{i}^{*}}^{2}}-\varepsilon^{2}\leq 0, \end{array}$$

(9c) $$\begin{array}{cc} \; & \frac{\sigma_{ij}\;\cdot \;\omega_{i}}{\omega_{j}}-1\leq 0, \end{array}$$

(9d) $$\begin{array}{cc} \; & Kx_{i}=b_{i}u, \end{array}$$

(9e) $$\begin{array}{cc} \; & \left(K-\omega_{i}^{2}M\right)\phi_{i}=0, \end{array}$$

(9f) $$\begin{array}{cc} \; & v=\frac{\sum_{e}\gamma_{e}v_{e}}{\sum_{e}v_{e}}\leq v^{*}, \end{array}$$

(9g) $$\begin{array}{cc} \; & 0<\gamma_{e}\leq 1. \end{array}$$

Here, we have defined the objective function (9a) as the maximization of static compliance of the structure, when a force is applied to the structure, e.g., actuation forces. The corresponding static force equilibrium is solved in (9d). We found that an energy-based objective function stabilizes the convergence of BESO. The optimization is then set up in such a way that the objective function becomes less influential compared to the frequency constraints, especially during the second optimization phase. Equations (9b)–(9f) define the constraints of the problem. (9b) enforces the frequencies of usable resonance modes $\omega_{i}$ to match the desired values $\omega_{i}^{*}$, while (9c) imposes a lower bound for undesired modes, often referred to as parasitic modes. It is defined as a ratio $\sigma_{ij}$ between a desired mode *i* and an undesired mode *j*. (9e) is the eigenvalue problem that needs to be solved during optimization. Finally, (9f) restricts the volume of the structure to a predefined value $v^{*}$ and $\gamma_{e}$ ranges from 0 to 1. For SIMP-based approaches, we enforce $\gamma_{e}>0$ by introducing $\gamma_{e,\min}=10^{-9}$ to avoid singularities.

In order to solve the optimization posed in (9), we use the well-established Lagrangian method. This extends the objective function (9a) to a Lagrangian function as follows:

(10) $$\begin{array}{c} \begin{array}{cc} L\left(\gamma ,\lambda ,s^{2}\right)=y+\lambda_{v}\left(\frac{\sum_{e}\gamma_{e}v_{e}}{\sum_{e}v_{e}}-v^{*}\right) & +\sum_{i}\lambda_{i}\left(\frac{{\left(\omega_{i}-\omega_{i}^{*}\right)}^{2}}{{\omega_{i}^{*}}^{2}}-\varepsilon^{2}+s_{i}^{2}\right)\\ \; & +\sum_{j}\lambda_{j}\left(\frac{\sigma_{ij}\;\cdot \;\omega_{i}}{\omega_{j}}-1+s_{j}^{2}\right), \end{array} \end{array}$$

with slack variables $s_{i/j}^{2}$ for the respective frequency constraints, turning them from inequality constraints into equality ones. $\lambda =(\lambda_{v},\lambda_{i},\lambda_{j})$ denotes the Lagrange multiplier for the respective constraint.

The sensitivity of the Lagrangian function (10) with respect to the design variables $\gamma_{e}$ is given by

(11) $$\begin{array}{c} \begin{array}{cc} \frac{\partial L}{\partial \gamma_{e}}=\sum_{i,j}x_{e,i/j}^{T}\frac{\partial K_{e}}{\partial \gamma_{e}}x_{e,i/j}+\lambda_{v}v_{e} & +\sum_{i}\lambda_{i}\left(\frac{2(\omega_{i}-\omega_{i}^{*})}{{\omega_{i}^{*}}^{2}}\;\cdot \;\frac{\partial \omega_{i}}{\partial \gamma_{e}}\right)\\ \; & +\sum_{j}\lambda_{j}\frac{\sigma_{ij}}{\omega_{j}^{2}}\left(\omega_{i}\frac{\partial \omega_{j}}{\partial \gamma_{e}}-\frac{\partial \omega_{i}}{\partial \gamma_{e}}\omega_{j}\right), \end{array} \end{array}$$

where $x_{e,i/j}$ denotes the displacement of element *e*’s nodes corresponding to mode *i* or *j*’s respective mode shapes. The derivatives of the frequencies are computed using Rayleigh quotients:

(12) $$\omega_{i/j}^{2}=\frac{\phi_{e,i/j}^{T}K\phi_{e,i/j}}{\phi_{e,i/j}^{T}M\phi_{e,i/j}},$$

where $\phi_{e,i/j}$ contains the component of the i/j-th eigenvector corresponding to the nodes of element *e*. This leads to

(13) $$\frac{\partial \omega_{i/j}}{\partial \gamma_{e}}=\frac{1}{2\omega_{i/j}}\phi_{e,i/j}^{T}\left(\frac{\partial K_{e}}{\partial \gamma_{e}}-\omega_{i/j}^{2}\frac{\partial M_{e}}{\partial \gamma_{e}}\right)\phi_{e,i/j}.$$

To solve the optimization problem, the solutions of the additional variables of the Lagrangian function are also required. Therefore, they are treated like design variables. The derivatives w.r.t. the Lagrange multipliers are the respective constraints—i.e.,

(14) $$\begin{array}{cc} \frac{\partial L}{\partial \lambda_{v}} & =\frac{\sum_{e}\gamma_{e}v_{e}}{\sum_{e}v_{e}}-v^{*}, \end{array}$$

(15) $$\begin{array}{cc} \frac{\partial L}{\partial \lambda_{i}} & =\frac{{\left(\omega_{i}-\omega_{i}^{*}\right)}^{2}}{{\omega_{i}^{*}}^{2}}-\varepsilon^{2}+s_{i}^{2}, \end{array}$$

(16) $$\begin{array}{cc} \frac{\partial L}{\partial \lambda_{j}} & =\frac{\sigma_{ij}\;\cdot \;\omega_{i}}{\omega_{j}}-1+s_{j}^{2}, \end{array}$$

and the derivatives with respect to the slack variables result in

(17) $$\frac{\partial L}{\partial s_{i/j}}=2\lambda_{i/j}s_{i/j}.$$

At an optimum, it is a necessary condition (Karush–Kuhn–Tucker) that all these derivatives (14)–(17) vanish. Therefore, with their help, an update scheme can be deployed for the search of these additional variables. In this work, we follow the update scheme suggested by [45,46]. As the domain of the additional variables introduced by the Lagrangian function is [0,∞), ref. [46] introduces a function to reduce the search space. They suggest mapping each of the Lagrange multipliers from [0,∞) to [0,1):

(18) $$\lambda_{i/j}=\frac{\mu_{i/j}}{1-\mu_{i/j}},\;0\leq \mu_{i/j}<1,$$

making them more compatible with the range of $\gamma_{e}$. Note that with a searching scheme, the Slack variables $s_{i/j}^{2}$ only serve as indicators for the respective constraint, indicating whether it is active or inactive. Therefore, they do not need to be determined [46].

## 4. Topology Optimization

**Figure 1:**
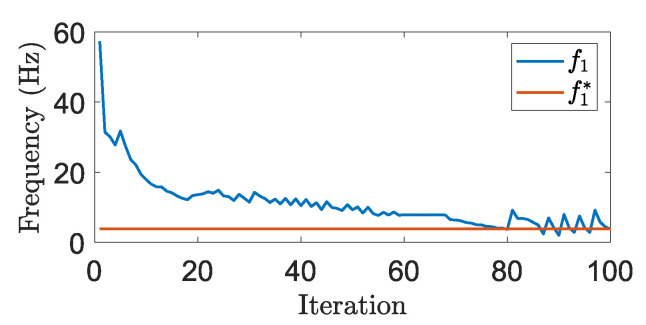
Convergence plot of the first (drive) frequency $f_{1}$ of the gyroscope, we consider in this work (see Section 5.1), during a BESO optimization [47]. Convergence issues, such as oscillation, are apparent towards the end of the optimization.

Topology optimization methods that are based on FE analysis mainly differ in their respective updating strategy. In [47], we applied BESO design multi-resonant MEMSs. Even though the optimizations resulted in promising designs, we experienced convergence issues with BESO. The resonant frequencies oscillate around the desired value during later iterations and sometimes even diverge (see Figure 1).

To remedy this issue, we suggest the introduction of a second optimization phase, in which a density-based approach is implemented to achieve algorithmic convergence. In this section, we illustrate the two TO methods applied in this work and propose a consecutive combination of both methods.

### 4.1. Bi-Directional Evolutionary Structural Optimization

**Figure 2:**
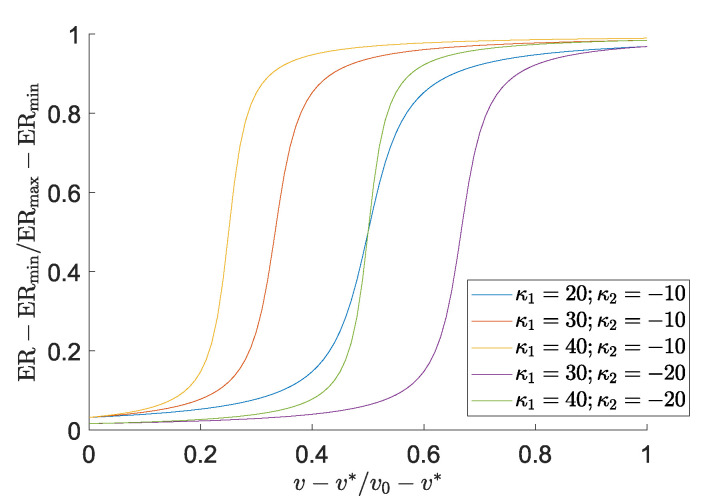
Evolutionary rate (ER) relative to maximum and minimum evolutionary rate (${\mathrm{ER}}_{\max},{\mathrm{ER}}_{\min}$) plotted versus volume fraction relative to desired volume fraction for some common $\kappa_{1}$ and $\kappa_{2}$ values that control changes in decrease evolutionary rate during optimization.

**Figure 3:**
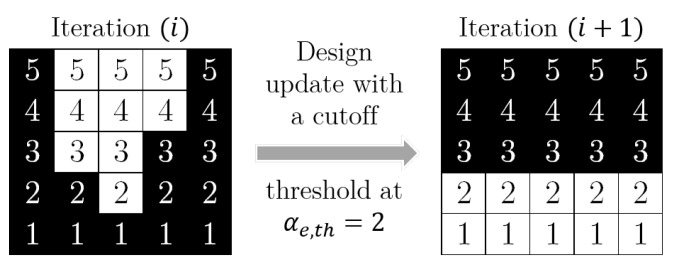
Diagram of the BESO update scheme. Black cells represent active and white cells represent inactive elements. The numbers correspond to the elemental sensitivity values $\alpha_{e}$. Elements with sensitivities $\alpha_{e}>\alpha_{e,th}$ are/remain activated and those with $\alpha_{e}\leq \alpha_{e,th}$ are deactivated.

The element updating rule of BESO deactivates elements with the lowest sensitivity values in each iteration. The deactivation threshold $\alpha_{e,th}$ is defined in every iteration and is derived from the dynamic evolutionary rate ER^(*i*)^, which specifies the number of elements remaining after each design update:

(19) $$\begin{array}{cc} {\mathrm{ER}}^{\left(i\right)}= & \frac{{\mathrm{ER}}_{\max}-{\mathrm{ER}}_{\min}}{\pi }\arctan\left(\frac{\kappa_{1}\left(v^{\left(i\right)}-v^{*}\right)}{v^{\left(0\right)}-v^{*}}+\kappa_{2}\right)+\frac{{\mathrm{ER}}_{\max}-{\mathrm{ER}}_{\min}}{2}, \end{array}$$

where ${\mathrm{ER}}_{\max}$ and ${\mathrm{ER}}_{\min}$ are the predefined upper and lower bounds for the evolutionary rate during optimization. $\kappa_{1}$ and $\kappa_{2}$ are parameters that control the location of the arctangent function’s inflection point and the function’s steepness around this point. Figure 2 shows the evolutionary rate ER versus volume fraction for some common $\kappa_{1}$ and $\kappa_{2}$ values in Figure 2. The update process is illustrated in Figure 3.

The sensitivity of an element w.r.t. compliance equals its strain energy [45], and the elemental sensitivity w.r.t. the resonance frequencies is derived in [48]. Coming from (11) and (13), the same sensitivities can be obtained by replacing the derivatives of the elemental matrices by

(20) $$\frac{\partial \{K_{e},M_{e}\}}{\partial \gamma_{e}}=\frac{\Delta \{K_{e},M_{e}\}}{\Delta \gamma_{e}}=\left\{K_{e},M_{e}\right\},$$

as elemental matrices vanish upon deactivation. The volume constraint is naturally considered in the updating scheme, so the corresponding part can be omitted, without affecting the order of the ranking:

(21) $$\alpha_{e}=\frac{\partial L}{\partial \gamma_{e}}-\lambda_{v}v_{e}.$$

To equal out the differences of the partial sensitivities induced by, e.g., the choice of units, we normalize the sensitivity values and project them onto the interval [0,1] (similar to [45]):

(22) $${\tilde{\alpha }}_{e}=\sum_{i,j}\frac{\alpha_{e,i/j}-\alpha_{i/j}^{\min}}{\alpha_{i/j}^{\max}-\alpha_{i/j}^{\min}},$$

where $\alpha_{e,i/j}$ refers to the part of the sensitivity corresponding to the *i*- or *j*-th constraint. The superscript max or min marks the maximum or minimum value of the partial sensitivity over all elements *e*. For example, let the objective function be maximizing the compliance of the first mode shape. In addition, the second resonance frequency should be optimized towards $\omega_{2}^{*}$, and the third resonance frequency should be higher than $2\;\cdot \;\omega_{2}$. Then, according to (21) and (11), the sensitivity is given by the following:

(23) $$\begin{array}{cc} \alpha_{e}= & \underset{=:\alpha_{e,1}}{\underset{︸}{\frac{1}{2}x_{e,1}^{T}K_{e}x_{e,1}}}+\underset{=:\alpha_{e,2}}{\underset{︸}{\lambda_{2}\frac{2(\omega_{2}-\omega_{2}^{*})}{{\omega_{2}^{*}}^{2}}\;\cdot \;\frac{\partial \omega_{2}}{\partial \gamma_{e}}}}+\underset{=:\alpha_{e,3}}{\underset{︸}{\lambda_{3}\frac{2}{\omega_{3}^{2}}\left(\omega_{2}\frac{\partial \omega_{3}}{\partial \gamma_{e}}-\frac{\partial \omega_{2}}{\partial \gamma_{e}}\omega_{3}\right)}}, \end{array}$$

with partial sensitivities $\alpha_{e,1}$ to $\alpha_{e,3}$. The normalization is applied to each of the partial sensitivities such that

(24) $$\begin{array}{c} {\tilde{\alpha }}_{e}=\frac{\alpha_{e,1}-\alpha_{1}^{\min}}{\alpha_{1}^{\max}-\alpha_{1}^{\min}}+\frac{\alpha_{e,2}-\alpha_{2}^{\min}}{\alpha_{2}^{\max}-\alpha_{2}^{\min}}+\frac{\alpha_{e,3}-\alpha_{3}^{\min}}{\alpha_{3}^{\max}-\alpha_{3}^{\min}}. \end{array}$$

It prevents partial sensitivities from being over-dominant and makes the overall sensitivity ill-conditioned.

Subsequently, we apply a filter to the sensitivities [49,50] to ensure the existence of solutions to the TO problem and to prevent commonly experienced issues, e.g., “checkerboard patterns” (e.g., [51,52]):

(25) $$\begin{array}{c} \begin{array}{c} {\hat{\alpha }}_{k}=\frac{\sum_{l\in N_{e,k}}w\left(r_{l}\right)\tilde{\alpha_{l}}}{\sum_{l\in N_{e,k}}w\left(r_{l}\right)},\\ w\left(r_{l}\right)=R_{\min}-\left|r_{k}-r_{l}\right|, \end{array} \end{array}$$

where $N_{e,k}$ is the set of adjacent elements in the neighborhood of element *k* and w(r) is the weight function based on a user-specified filter radius $R_{\min}$ and the Euclidean distance between the elements *k* and *l* ($r_{k}$ and $r_{l}$ denote the coordinates of the center point of the respective element). This sensitivity filtering is effectively a blurring of the values using a hat function. Therefore, this operation enables sensitivity values to be assigned to an inactive element, allowing it to become active again.

The reactivation of elements is further encouraged by the final modification. In [53], possible convergence issues were reported for evolutionary TO, such as jumps or the oscillation of objective values. One reason for these issues is the inaccurate sensitivities of the inactive elements, which may not be present in the FE-Model. Therefore, the authors suggested relaxing the difficulty of convergence by introducing history-averaging that smoothens the change sensitivity values from the (i−1)-th to the (i)-th TO iteration:

(26) $${\bar{\alpha }}_{e}=\frac{1}{2}\left({\hat{\alpha }}_{e}^{\left(i\right)}-{\hat{\alpha }}_{e}^{\left(i-1\right)}\right).$$

A major challenge during BESO is to conserve the structural integrity of the layout, i.e., to ensure that the layout does not become disconnected. Many studies have been published in recent years tackling this issue, e.g., [54]. The current implementation uses a similar approach to the aforementioned publication. Instead of a connectivity matrix, we use image recognition to check for the number of bodies present in each iteration. In case there is more than one body present during the check, a test identifies the elements causing the loss of connection. These elements (and their neighbors) are then excluded from optimization. This approach can also be used to control the minimum length scale present in the structure if a minimum number of adjacent elements is required for each element.

### 4.2. Density-Based Approach

For the second phase, the traditional simple isotropic material with penalization (SIMP) is implemented for the interpolation of material properties:

(27) $$\begin{array}{c} \begin{array}{cc} E_{e} & =E_{\min}+\gamma_{e}^{p}\left(E_{\mathrm{full}}-E_{\min}\right),\\ \rho_{e} & =\rho_{\min}+\gamma_{e}\left(\rho_{\mathrm{full}}-\rho_{\min}\right). \end{array} \end{array}$$

$E_{\mathrm{full}}$ and $\rho_{\mathrm{full}}$ denote the Young’s modulus and density of the material and $E_{\min}=10^{-9}\;\cdot \;E_{\mathrm{full}}$ and $\rho_{\min}=10^{-9}\;\cdot \;\rho_{\mathrm{full}}$ are the respective values assigned to void elements, as $E_{\min}=\rho_{\min}=0$ would lead to singular stiffness and mass matrices. *p* is the penalty factor, which is usually set to three by default (see [55] for a more in-depth discourse on the SIMP approach and the choice of the penalty factor).

For the density-based approach, we adopt the projection scheme from [56]. Here, the blurring filter is applied to the design variable instead of the sensitivity:

(28) $$\begin{array}{c} \begin{array}{c} {\hat{\gamma }}_{k}=\frac{\sum_{l\in N_{e,k}}w\left(r_{l}\right)\tilde{\gamma_{l}}}{\sum_{l\in N_{e,k}}w\left(r_{l}\right)},\\ w\left(r_{l}\right)=R_{\min}-\left|r_{k}-r_{l}\right|. \end{array} \end{array}$$

**Figure 4:**
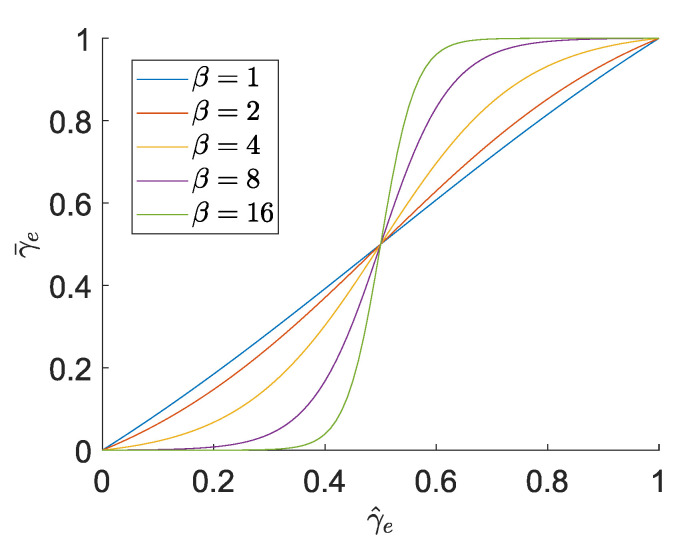
Heaviside function used for threshold projection from (29) for different values of β. The threshold η=0.5 sets the inflection point of the function. Intermediate values ${\hat{\gamma }}_{e}<\eta$ are pushed towards 0 and ${\hat{\gamma }}_{e}>\eta$ towards 1; the higher β is, the further the values are pushed.

This allows us to introduce a subsequent threshold projection using a smoothed Heaviside function [56] to suppress gray elements, i.e., elements with intermediate density $0<{\hat{\gamma }}_{e}<1$:

(29) $${\bar{\gamma }}_{e}=\frac{\tanh\left(\beta \eta \right)+\tanh(\beta \left({\hat{\gamma }}_{e}-\eta \right))}{\tanh(\beta \eta )+\tanh(\beta (1-\eta ))},$$

where η is the threshold, e.g., an element with $\hat{\gamma }<\eta$ will be pushed towards being a void element and vice versa. The “aggressiveness” of the filter is controlled by the sharpness parameter β, which determines the steepness of the slope in the Heaviside function. The Heaviside function for different values of β and η=0.5 is plotted in Figure 4.

For the sake of consistency, the Lagrangian function (10) is also considered for the density-based TO. Therefore, the initial sensitivity is identical to (11), with

(30) $$\begin{array}{c} \begin{array}{c} \frac{\partial K_{e}}{\partial \gamma_{e}}=p\gamma_{e}^{p-1}K_{e}\;\mathrm{and}\;\frac{\partial M_{e}}{\partial \gamma_{e}}=M_{e}, \end{array} \end{array}$$

due to the SIMP interpolation scheme. This, then, has to be modified with the terms introduced by the applied filters and the chain rule:

(31) $$\begin{array}{cc} \frac{\partial {\hat{\gamma }}_{k}}{\partial \gamma_{l}}= & \frac{w(r_{l})}{\sum_{l\in N_{e,l}}w\left(r_{l}\right)}, \end{array}$$

(32) $$\begin{array}{cc} \frac{\partial {\bar{\gamma }}_{e}}{\partial {\hat{\gamma }}_{e}}= & {\left[\beta \cosh^{2}\left(\beta \left({\hat{\gamma }}_{e}-\eta \right)\right)\;\cdot \;\left[\tanh(\beta \eta )+\tanh(\beta (1-\eta ))\right]\right]}^{-1}, \end{array}$$

where (31) corresponds to the blurring filter and (32) corresponds to the threshold projection. In total, the sensitivity of an element *e* is determined by

(33) $$\alpha_{e}=\sum_{l\in N_{e,e}}\frac{\partial L}{\partial \bar{\gamma_{l}}}\frac{\partial {\bar{\gamma }}_{l}}{\partial {\hat{\gamma }}_{l}}\frac{\partial {\hat{\gamma }}_{l}}{\partial \gamma_{e}}.$$

Furthermore, we also adopted the normalization introduced in (22). However, as opposed to BESO, not only does the relative ranking of the elemental sensitivities matter, but also their respective signs are required for density-based TO. Therefore, the normalization is adjusted to

(34) $${\tilde{\alpha }}_{e}=\sum_{i,j}\frac{\alpha_{e,i/j}}{\left|\right|\alpha_{i/j}{\left|\right|}_{\infty }},$$

with $\alpha_{e,i/j}$ again being the part of the sensitivity corresponding to the *i*- or *j*-th constraint. $\left|\right|\alpha_{i/j}{\left|\right|}_{\infty }$ is the infinity norm of the vector $\alpha_{i/j}$, which contains all $\alpha_{e,i/j}$ of all elements.

### 4.3. Combining BESO with SIMP and MOR

The combined approach is defined as follows: at the beginning, BESO is initiated with a full design space. The discrete optimization is executed until the target volume fraction is achieved. Subsequently, we switch to the density-based approach. In this phase, we also introduce projection-based MOR to minimize the computational effort required during iterations. As the resonance frequencies (and therefore, the eigenvectors) need to be computed in every iteration, one could opt for a modal reduction of the model without any additional efforts required. Due to the pre-optimization with BESO, we do not expect large changes in structure; therefore, the projection basis *V* can be reused over a large number of iterations. This would make, e.g., Krylov subspace-based MOR or balanced truncation feasible for static objective functions.

**Figure 5:**
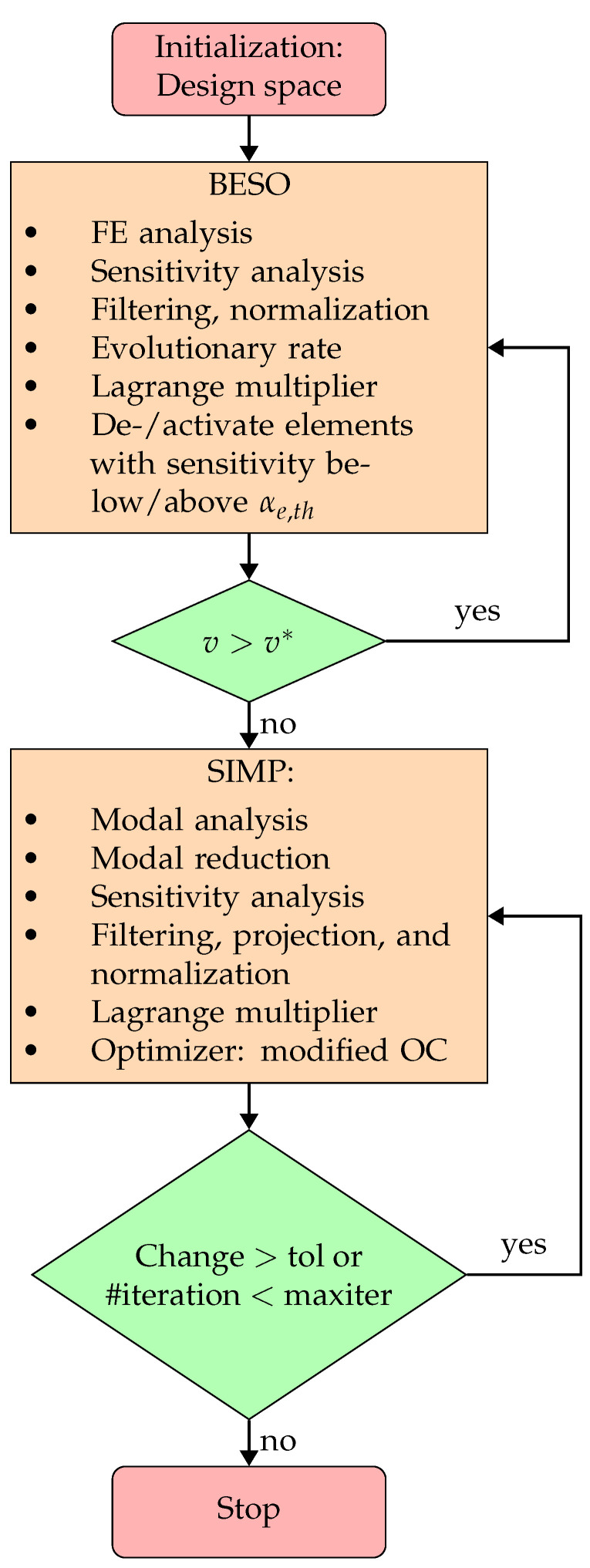
Flow chart of proposed two-phase TO approach using modal reduction and modified OC as optimizer.

As suggested in Section 4.1 and Section 4.2, the Lagrangian function is kept throughout both optimization phases for consistency. The Lagrange multipliers are determined with (18), where the supplement variables $\mu_{i/j}$ are initialized with small values and subsequently increased throughout both phases as long as the corresponding constraint is violated. A flowchart of the proposed TO approach is shown in Figure 5.

Note that we do not impose any geometric or gray penalization constraints as suggested in [34]. In our combined approach, the related issues are naturally taken care of by the BESO implementation. In our test cases, thin structures are exclusively generated during BESO optimization. Therefore, no “gray connections” (see [34]) need to be prevented. A threshold projection is still required to suppress gray areas. However, due to the BESO pre-optimization, the filter can be initialized at a higher β value.

## 5. Optimization Results

In this section, we apply the proposed combined TO approach to two resonant MEMS structures. Both design spaces are implemented in Ansys^®^ Mechanical Enterprise Academic Research, Release 2023.1 [57]. TO is performed using pyAnsys, Release 2023.1.3 [58]. Both models are discretized using linear elements with an element size of two (μm). A detailed overview of optimization parameters used for each benchmark will be presented in the respective subsection. The optimal layouts are presented and discussed. Additionally, a comparison of the two-phase approach to BESO-only and purely density-based TO, as well as with the reference source, is provided for the first benchmark.

### 5.1. Benchmark: MEMS Gyroscope

**Figure 6:**
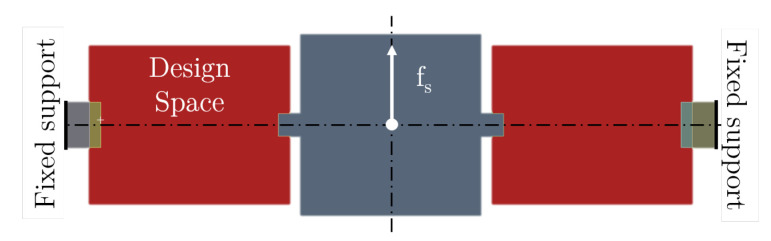
Static load case to emulate the displacement of the proof mass in the third (sense) mode shape. The compliance of this load case is used as an objective function to maximize the amplitude of the sense mode.

In the first benchmark, we apply our combined approach to a simplified academic test case proposed in [34]. The considered structure is an in-plane linear MEMS gyroscope. Gyroscopes are inertial sensors for angular acceleration or velocities, making use of the Coriolis effect. For more details on MEMS gyroscopes, please refer to, e.g., [59]. Adopted from the reference paper, we target a drive frequency of $f_{1}=3.8$, a sensing frequency of $f_{2}=4$, and a frequency for the parasitic mode beyond $f_{3}>1.4\;\cdot \;f_{2}$. Instead of formally maximizing the sense response, we choose to maximize the compliance of the structure for the load case depicted in Figure 6. Low-frequency motions of MEMS structures with a concentrated proof mass and light suspending structures are dominated by the motion of the proof mass (a similar assumption is applied in [34]. Therefore, it is sufficient to impose a single force onto the mass’ center of gravity. In this way, we aim to maximize the deformation of the structure caused by the Coriolis force, which serves as a simplified estimation for the sense response. This is a deliberate choice, as compliance-based objective functions are applicable for a wide range of MEMS devices, whereas the sense response due to Coriolis force is very specific to gyroscope design.

**Figure 7:**
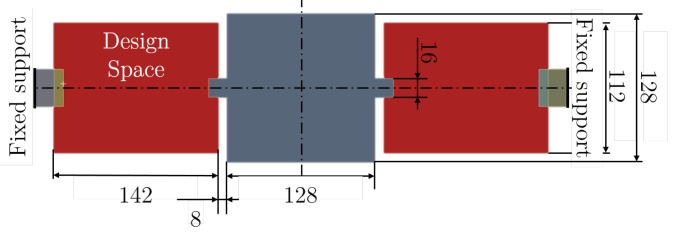
Design space and boundary conditions for the benchmark: MEMS gyroscope.

The two-dimensional design space of this benchmark is depicted in Figure 7. The design space and the material parameters are both taken from the reference paper. The square proof mass has a dimension of 128×128. The design space on both sides of the proof mass is sized at 142×112. As described in the introduction, the element size is set to two. The element size of the model is increased compared to the reference paper due to instability in the communication between Python 3.9 and Ansys. To avoid undesired interaction between boundaries and filters, boundary blocks and a buffer zone are introduced [60]. A unitary isotropic material is assumed with Young’s modulus E=1, Poisson’s ratio ν=0.3, and a density of $\rho =1.76\;\cdot \;10^{-10}$ (see Table 1).

For BESO, we set the upper and lower limits of the evolutionary rate to ${\mathrm{ER}}_{\min}=0.005$ and ${\mathrm{ER}}_{\max}=0.05$. The parameters $\kappa_{1}$ and $\kappa_{2}$ are chosen as 40 and −20, respectively. The desired final volume fraction of the structure is set to $v^{*}=0.422$, adopted from the reference paper. The Lagrange multipliers are initialized with $\lambda_{i/j}=0$. Upon violation of the constraint *i* or *j*, $\lambda_{i/j}$ is increased to one—i.e., the corresponding auxiliary parameters $\mu_{i/j}$ are set to 0.5 and then increased by 0.004 after each iteration, as long as the constraint remains violated with a tolerance of ε=0.025. For filter parameters, the filter radius of the blurring filter used in this experiment is $R_{\min}=8$ (reference: $R_{\min}=4$). This is a consequence of the increased element size. In the threshold projection, we set $\eta =v^{*}$ and β is initialized with β=8, which is then increased linearly during the second phase. The increase is set to 2 every 20 iterations up to β=16.

To reduce computation time, we exploit the symmetry of the design space and only perform TO on one of the quadrants (here, we use the bottom left quadrant). Additionally, a modal projection base, composed of the first three eigenvectors of the dynamical system is used for model order reduction during the SIMP phase. This means that only three linear equations need to be solved during each optimization loop (see Section 5.3). Finally, the modified optimality criteria method proposed in [61] is implemented as an optimizer. The change tolerance for convergence is set to 1% and the maximum number of iterations performed with the density-based method is restricted to 120.

**Figure 8:**
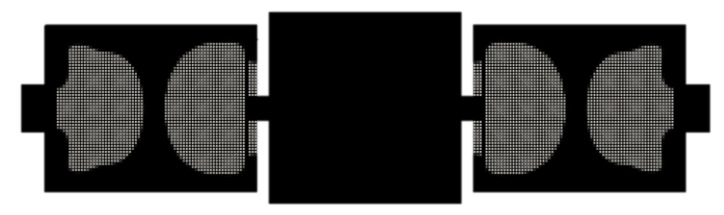
Optimized layout for the benchmark: MEMS gyroscope. The volume fraction of the final structure is $v^{*}=0.422$. Its mode shapes and their respective frequencies are presented in Figure 9. Full and void elements are black and light gray, respectively.

**Figure 9:**
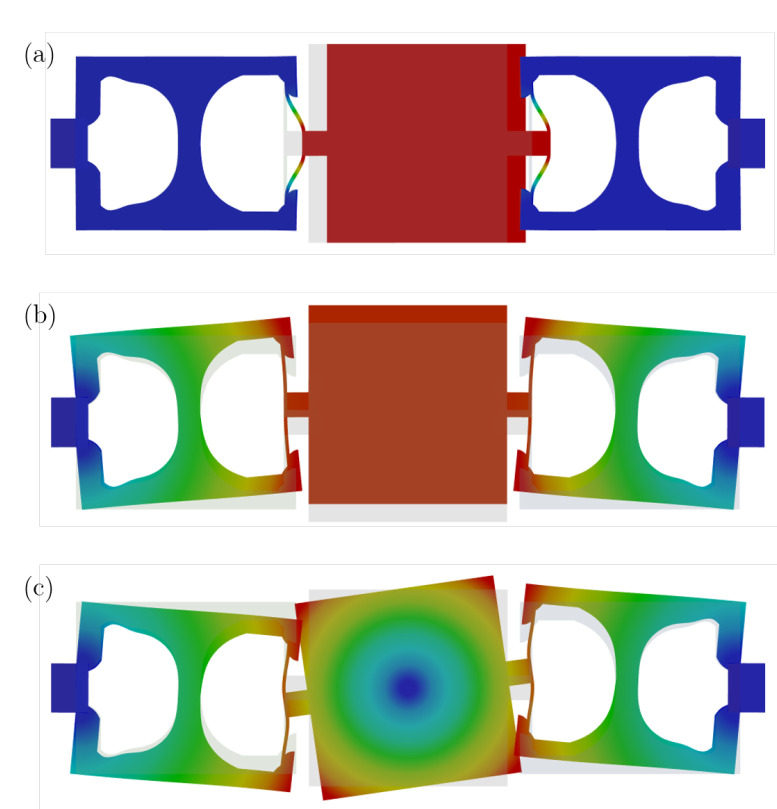
Mode shapes of the optimized MEMS gyroscope in Figure 8. (**a**) First mode shape (drive mode—frequency 3.77). (**b**) Second mode shape (sense mode—frequency 4.05). (**c**) Third mode shape (parasitic mode—frequency 5.49).

The optimized layout is presented in Figure 8. A comparison of the achieved resonance frequencies versus the target values is listed in Table 2. Figure 9 shows the mode shapes of the optimized structure.

#### Comparison of Algorithms

In this section, we compare the results of the combined method with the results of BESO and density-based TO. For this comparison, we set up all optimizations with identical objective functions and constraints. The settings for both algorithms are identical to those deployed in the two-phase algorithm if possible. For density-based TO, we replaced the BESO pre-optimization by initially setting the filter parameter to β=1. β is then doubled every 20 iterations until β=8. After that, β is increased linearly, identical to the two-phase approach. With this configuration, the maximum number of iterations is identical across all optimizations. Additionally, we introducd the gray penalization constraints suggested by [34],

(35) $$\begin{array}{c} 1-\frac{\omega_{d}^{\left(*\right)}}{\alpha_{\mathrm{gray}}\;\cdot \;\omega_{d}}\leq 0, \end{array}$$

(36) $$\begin{array}{c} 1-\frac{\omega_{s}^{\left(*\right)}}{\alpha_{\mathrm{gray}}\;\cdot \;\omega_{s}}\leq 0, \end{array}$$

to ensure the integrity of the structure. Here, $\omega_{d}^{\left(*\right)}$ and $\omega_{s}^{\left(*\right)}$ are the drive and sense frequencies of the structure derived from the same set of intermediate design variables (before filtering) but with a more strict Heaviside filter, i.e., $\eta^{\left(*\right)}=2\eta$ in our case. $\alpha_{\mathrm{gray}}=0.35$ is adopted for this comparison.

**Figure 10:**
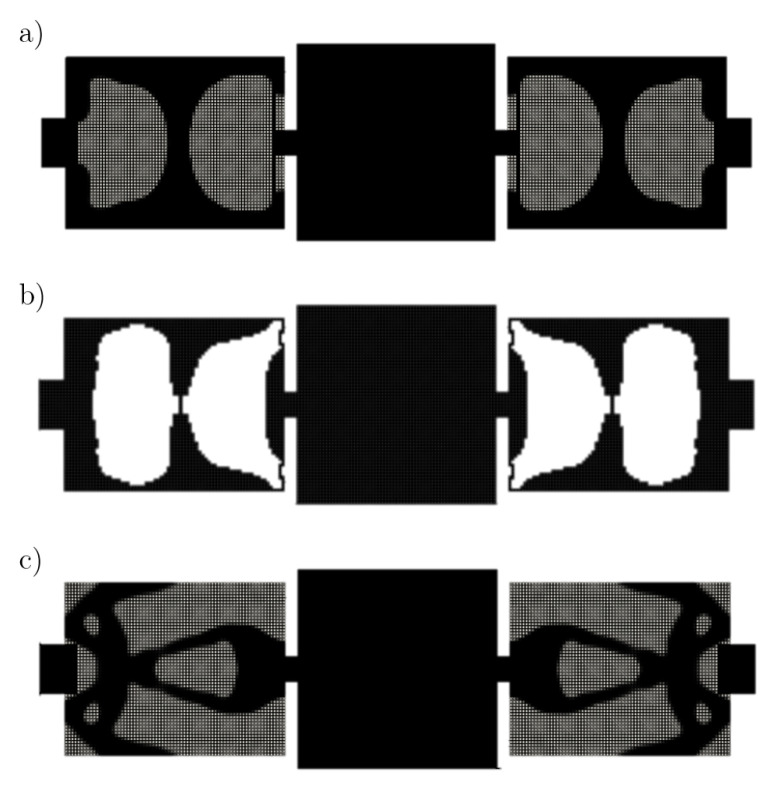
Comparison of the optimized geometries obtained by (**a**) two-phase optimization, (**b**) BESO, and (**c**) density-based TO with identical objective functions and constraints. The frequencies of the respective structures are compared in Table 3.

The BESO-only approach resulted in a layout with some thin (folded) flexural beam structures, which closely resemble those utilized in classical MEMS layouts (see Figure 10b). Unfortunately, most of these structures disappear during the second phase, when combining both methods. The optimized geometry obtained by a purely density-based method resembles the structure from [34], which was also obtained by a purely density-based method. For comparison, the layouts in [34] are achieved with numbers of iterations ranging from 1232 to 1703. We capped the total number of iterations at 180 for all optimizations. This is due to the aforementioned issues concerning communication instability when interfacing with the commercial software. Consequently, the imposed tolerances for the target frequencies were increased from $3\;\cdot \;10^{-4}$ to 0.025 and the filter parameters also increased more rapidly. We do not claim and never expected the proposed, generic approach to outperform the reference paper, even with a similar number of iterations. However, this comparison with a well-tuned TO, specifically designed for this kind of device, clearly shows the potential and deficit of the approach.

More specifically, the comparison shows that BESO exhibits faster convergence towards optimal designs. We mainly attribute this to BESO not requiring complex filtering techniques. However, as stated in [62], BESO tends to converge towards inefficient local optimal designs, which is also apparent in this comparison. On a positive note, we can report that the inclusion of a second optimization phase successfully eliminated the convergence issues we encountered during BESO-only optimization. However, further investigation is required into whether a higher number of iterations with the density-based methods would lead to a recovery from the local optimum achieved by BESO for more efficient designs.

### 5.2. Benchmark: MEMS Scanning Mirror

**Figure 11:**
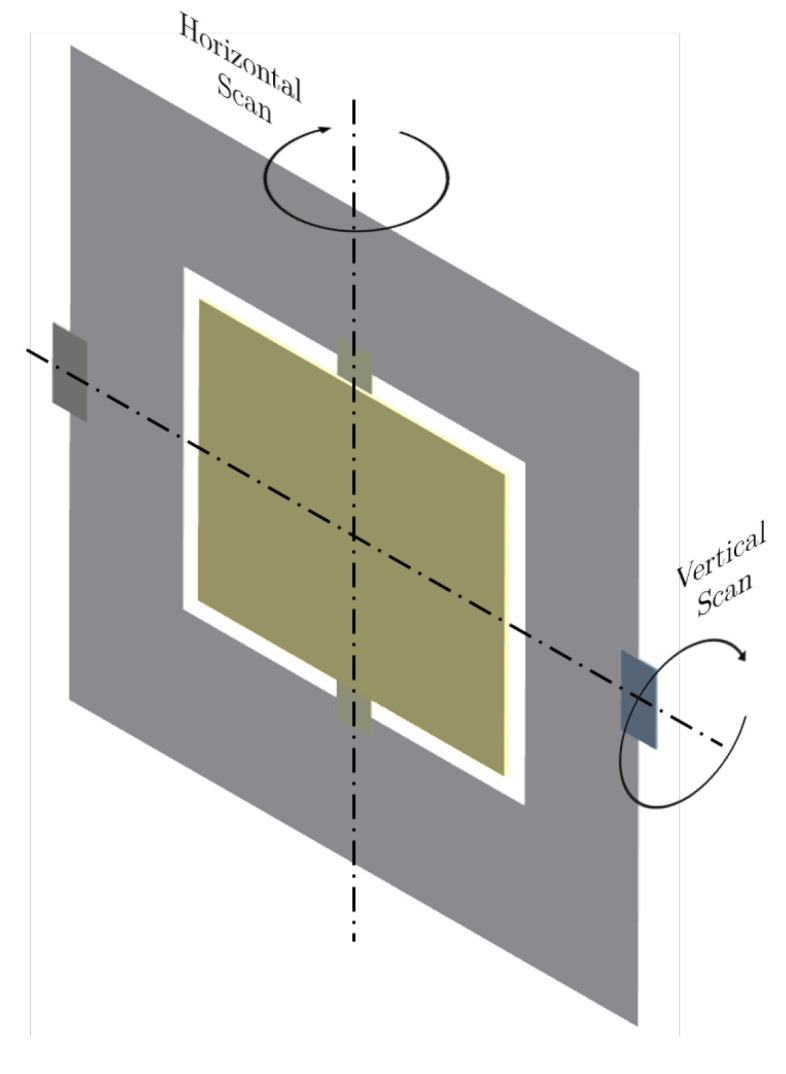
Schematic of the micromirror showing the two rotational degrees of freedom for horizontal and vertical scanning.

**Figure 12:**
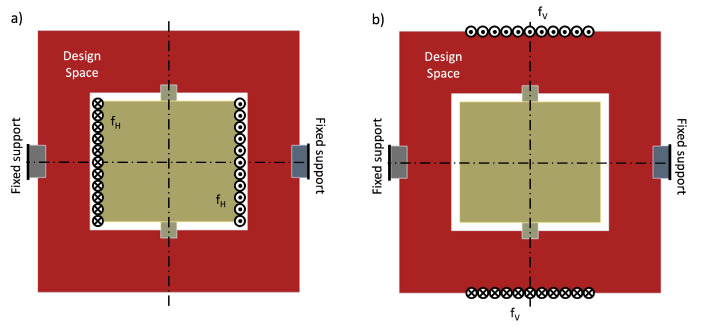
Static load cases to emulate the actuation of the mirror in (**a**) horizontal scanning motion and (**b**) vertical scanning motion. The compliance of these load cases is used as an objective function to maximize the deflection angle.

In this benchmark, we apply our combined approach to a second, more realistic MEMS device, with actual dimensions and material properties. The device is an MEMS scanning mirror, often also referred to as a micromirror, with two rotational degrees of freedom. The tilting motion of the mirror, as shown in Figure 11, can be utilized to control the direction of reflected light rays. This feature is utilized in laser scanner projection applications. The typical performance parameters of an MEMS scanning mirror are its maximum scan angle, resonant frequency, resolution, and surface quality, i.e., smoothness and flatness [63]. In this benchmark, our approach is used to design a micromirror for image projection at 60 Hz and a scanning resolution of 1080 horizontal lines. According to the desired performance parameters, we set the objective function to maximize the amplitude of both resonance modes to maximize their respective deflection angle. The objective function is set as the sum of compliance over the static load case depicted in Figure 12. In this way, we aim to maximize the horizontal deflection angle during operation. Note that a resonance frequency of 60 Hz is not feasible for this considered design space due to its dimensions and the involved material. As a consequence, the vertical scanning motion will be actuated outside of resonance. Therefore, we minimize the frequency, which minimizes the energy required for actuation. In contrast, the horizontal scanning motion is driven in resonance; thus, we set the target frequency for the horizontal scanning mode to 1080×60 = 64,800 Hz.

**Figure 13:**
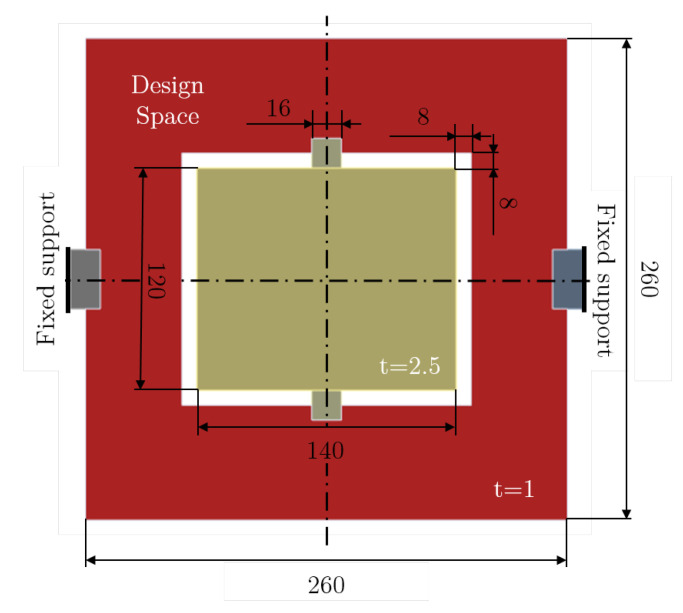
Design space and boundary conditions for the benchmark: MEMS scanning mirror. All dimensions are given in micrometers.

The design space considered for this optimization is depicted in Figure 13. The mirror in the center of the structure has a dimension of $140\times 120\times 2.5\;\mu^{3}m$ similar to the structure proposed in [64]. The flexible structure surrounding the mirror is defined as the design space and has a dimension of $260\times 260\times 1\;\mu^{3}m$. Also here, boundary blocks and buffer areas are introduced to avoid undesired boundary interactions. The material parameters are Young’s modulus E=169GPa, Poisson’s ratio ν=0.3, and density $\rho =2330\;kg/m^{3}$ (see Table 4).

For the BESO, we reduced the limits of the evolutionary rate to ${\mathrm{ER}}_{\min}=0.008$ and ${\mathrm{ER}}_{\max}=0.01$. $\kappa_{1}=40$ and $\kappa_{2}=-20$ are kept from the gyroscope benchmark. The final volume fraction is set to 0.5. Again, the Lagrange multipliers are initialized with $\lambda_{i/j}=0$, and upon constraint violation, they and their auxiliary parameters $\mu_{i/j}$ are increased to 1 and 0.5, respectively. Here, the subsequent increase is changed to 0.01 after each iteration until the constraint becomes inactive within a tolerance of ε=0.01. All filter parameters remain identical to those used for the first benchmark, i.e., $R_{\min}=8$, η=0.5, and β=8⋯16. The same applies to the optimizer, maximum number of iterations, and convergence tolerance. Finally, quarter symmetry is again assumed, and the same “three-modes” modal reduction is performed to speed up the SIMP phase (see Section 5.3).

**Figure 14:**
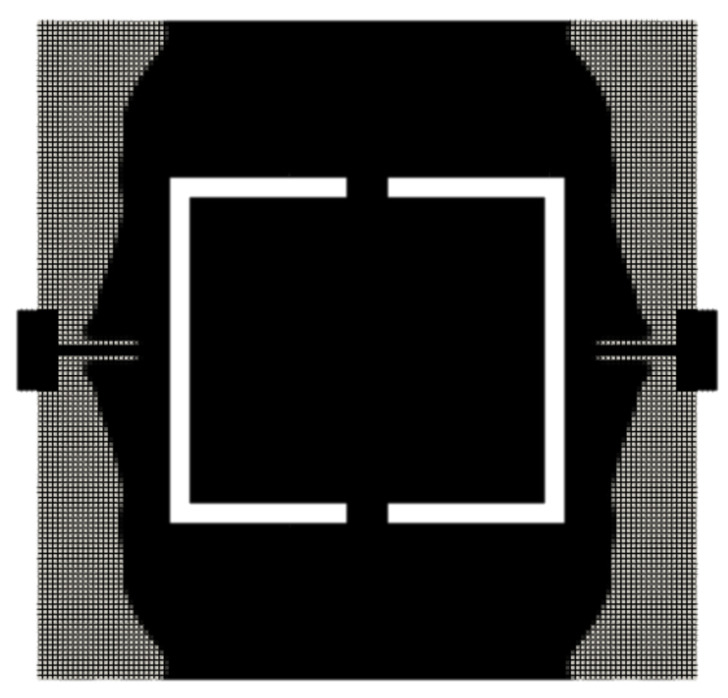
Optimized layout for the benchmark: MEMS scanning mirror. The volume fraction of the final structure is $v^{*}=0.5$. Its mode shapes and their respective frequencies are presented in Figure 15. Full and void elements are black and light gray, respectively. White buffer areas are not part of the meshed model.

**Figure 15:**
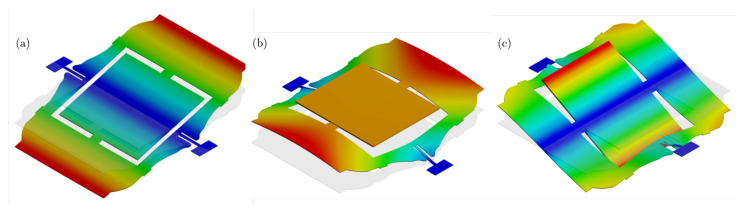
Mode shapes of the optimized MEMS scanning mirror in Figure 14. (**a**) First mode shape (vertical scanning mode—frequency 12,022 Hz). (**b**) Second mode shape (parasitic mode—frequency 26,302 Hz). (**c**) Third mode shape (horizontal scanning mode—frequency 64,147 Hz).

The optimized layout is presented in Figure 14. Table 5 shows the resonance frequencies of the optimized layout and the corresponding objective for the respective modes, and Figure 15 depicts the mode shapes of the optimized structure.

The optimized geometry achieved a layout satisfying the objectives and constraints defined in the optimization problem. To minimize the first resonance frequency, the material is concentrated at the top and bottom of the layout, and thin flexural beams are formed extending from the fixed support. These resemble features present in published designs, e.g., [65]. However, it is also evident that, if feasible, increasing the size of the design space would greatly benefit the optimization for the given objectives. Additional mass and an increasing length of the thin flexural structure would allow the optimizer to further reduce the resonance frequency of vertical scanning. Alternatively, an increase in thickness may also benefit the frequency characteristics. It would also allow for a connection between the mirror and design space with a reduced width, without compromising on the resonance frequency for horizontal scanning. Reducing the width of the connection would improve the planarity of the mirror during operation.

### 5.3. Model Order Reduction

As stated in Section 5.1 and Section 5.2, model order reduction is introduced to alleviate the computational burden. The model is reduced in such a way that the reduced-order model (ROM) only contains four degrees of freedom (DoFs). This is considerably lower in dimension compared to the full-order model (FOM) originating from FE modeling. The computation times required for the TO of both benchmark devices with and without the inclusion of MOR are shown in Table 6. For this comparison, we only consider the net computation time required for the second phase of the optimization process. This excludes, i.e., all file input and output and the overhead generated by the interaction between Python and Ansys. Table 6 shows a significant reduction in computation time when MOR is involved. The computation time required for the optimization of the gyroscope is reduced by half. The reduction in computation time achieved during the optimization of the micromirror is 66%. This demonstrates the potential of MOR-aided TO.

## 6. Conclusions and Outlook

In this work, we investigated the feasibility of a two-phase approach for a fast topology optimization of multi-resonant MEMS applications. The approach includes an initial optimization using BESO and a subsequent SIMP-based TO to achieve convergence. To further reduce the computation time, we introduced modal-based MOR during the second phase.

The proposed approach was applied to the design of two different benchmark models. In the first benchmark, we applied the approach to a single-mass, in-plane MEMS gyroscope. The optimized layout presented in Section 5.1 achieved the desired target resonance frequencies within the given tolerances. The comparison with BESO-only and purely density-based TO shows the following:

1. The convergence issues of BESO can be circumvented with the inclusion of a subsequent, density-based optimization;
2. BESO requires fewer iterations to converge to optimal designs …;
3. However, BESO tends towards local, inefficient optima that may not be recovered with a low number of density-based iterations.

In a second benchmark, we applied our two-phase approach to the design of a micromirror with two rotational degrees of freedom. The optimized layout was designed for imaging projection, with a resolution of 1920×1080 at 60 Hz. The proposed approach converged to a layout fulfilling the posed requirements. Depending on the spatial availability in the given application, a dimensional increase of the design space may further improve the design. Note that both models are generic test models. For specific applications, there are typically additional performance indicators or constraints to be considered, e.g., rigidity for specific disturbances or fabrication constraints. These, however, can be easily considered during optimization through additional terms in the objective function or as additional constraints.

The inclusion of MOR significantly reduced the computation time required for the second phase. We expect the advantage of MOR to further increase when the goal function becomes more complex. This is because MOR only affects the objective function. However, the optimization also includes many processes that do not benefit from MOR, such as the computation of the mode shapes and the resonance frequencies. Therefore, an optimized implementation of the method will also increase the advantage of MOR, as this would reduce the overall computation time, particularly the time required by the processes not affected by MOR.

During our experiments, we did not experience any change in convergence or final optimized layout due to MOR for both of the presented benchmarks. However, this needs further investigation. Future research may also exploit AI-based prediction for objective values such as resonance frequencies or compliance to achieve even faster optimizations: In [66], a neural network is trained to predict the resonant frequency, thermoelastic quality factor, and other quality measures of an MEMS resonator that can be relevant for design optimization. The prediction is significantly faster than traditional numerical simulation while delivering similarly accurate results.

Another relevant issue that shall be addressed in future research is the preservation of the structural integrity of the layout. The current implementation requires significant computational effort. The restore and exclude mechanism designed to prevent the layout from becoming disconnected makes the BESO process very sensitive to the change of optimization parameters, and the final layout is dependent on the course or history of the optimization. A more sophisticated approach could be, e.g., describing the FE mesh as a weighted graph, with elemental sensitivities as weights. Then, one could implement a path-finding algorithm such as A* [67] to find the shortest path along elements w.r.t. these sensitivities in each iteration. This would be a more deterministic approach and ensure structural integrity at minimal cost w.r.t. the optimization problem.

## Figures and Tables

**Table 1:** Parameters of the unit isotropic material used to model the MEMS gyroscope.

Young’s Modulus *E*	Poisson’s Ratio ν	Density ρ
1	0.3	$1.76\;\cdot \;10^{-10}$

**Table 2:** Benchmark: MEMS gyroscope. Resonance frequencies of the optimized layout versus their respective target values.

Mode	Frequency	Target Frequency
Drive mode	3.77	3.8
Sense mode	4.05	4.0
Parasitic mode	5.49	5.6

**Table 3:** Benchmark: MEMS gyroscope. Comparison of the frequencies achieved by the two-phase approach, BESO, and density-based TO.

Mode	2-Phase	BESO ^†^	SIMP	Target Freq.
Drive	3.77	4.05	5.31	3.8
Sense	4.05	4.51	5.34	4.0
Parasitic	5.49	6.00	8.79	5.6

^†^ As shown in Figure 1, BESO exhibits convergence issues, i.e., oscillation of the resonance frequencies around the desired value. The best result is achieved after 80 iterations.

**Table 4:** Parameters of the isotropic polysilicon material used to model the MEMS scanning mirror.

Young’s Modulus *E*	Poisson’s Ratio ν	Density ρ
169 GPa	0.3	2330 $kg/m^{3}$

**Table 5:** Benchmark: MEMS micro scanning mirror. Resonance frequencies of the optimized layout versus their respective target values.

Mode	Frequency	Target Freq.
Vertical scanning mode	12,022 Hz	$\min f_{1}$
Parasitic mode	26,302 Hz	n/a ^‡^
Horizontal scanning mode	64,147 Hz	64,800 Hz

^‡^ The parasitic mode is not relevant in this case, as the vertical scanning mode will be actuated and thus suppress the parasitic mode.

**Table 6:** Comparison of computation time required for both benchmark devices’ TO process with and without MOR. The optimization was performed on an Intel^®^ CORE™ i9-9900X CPU @ 3.5 GHz with 64 GB RAM.

Benchmark	DoF ^**1**^	Computation Time ^**2**^
Gyroscope	62,265 (FOM)	1677.95 s
	4 (ROM)	773.74 s
Micromirror	83,043 (FOM)	3236.42 s
	4 (ROM)	1106.86 s

^1^ The model’s degrees of freedom, i.e., model dimension minus degrees of freedom, were removed due to imposed Dirichlet boundary conditions. ^2^ We only considered the net computation time required for SIMP optimization. This excludes, i.e., input/output from/to the hard drive or the interface time between Python and Ansys.

## Data Availability

The manuscript provides all the information needed by readers to replicate the presented results. The authors have stored all data related to the presented examples and ensured that all relevant parameters are stated clearly throughout the manuscript. Furthermore, the code has been uploaded to Github and can be made available upon request to the corresponding author.

## Abbreviations

The following abbreviations are used in this manuscript:

AI	Artificial Intelligence
(B)ESO	(Bidirectional) Evolutionary Structural Optimization
DoF	Degrees of Freedom
FE	Finite Element
FOM	Full-Order Model
MEMS	Microelectromechanical system
MOR	Model Order Reduction
OC	Optimality Criteria
POD	Proper Orthogonal Decomposition
ROM	Reduced-Order Model
SIMP	Solid Isotropic Material with Penalization
SVD	Singular Value Decomposition
TBR	Truncated Balanced Realization
TO	Topology Optimization
